# Cloning and expression of SOLD1 in ovine and caprine placenta, and their expected roles during the development of placentomes

**DOI:** 10.1186/1471-213X-10-9

**Published:** 2010-01-21

**Authors:** Koichi Ushizawa, Toru Takahashi, Misa Hosoe, Keiichiro Kizaki, Kazuyoshi Hashizume

**Affiliations:** 1 Reproductive Biology Research Unit, Division of Animal Sciences, National Institute of Agrobiological Sciences, 2 Ikenodai, Tsukuba, Ibaraki 305-8602, Japan; 2 Department of Veterinary Medicine, Faculty of Agriculture, Iwate University, 3-18-8 Ueda, Morioka, Iwate 020-8550, Japan

## Abstract

**Background:**

The Ly-6 (Ly-6/uPAR) superfamily members share the Ly-6 domain defined by distinct disulfide bonding patterns between 8 or 10 cysteine residues. They comprise membrane- and secretory-type proteins. We recently reported the gene and protein characterization of the bovine secreted protein of Ly-6 domain 1 (SOLD1). Bovine SOLD1 is expressed in trophoblast mononucleate cells (TMCs) and is localized in the cotyledonary mesenchyme. Here, we compared the expression and functionality of SOLD1 among the ruminants. We examined mRNA expression by chorionic fibroblasts as a measure of one of the SOLD1 functions.

**Results:**

Ovine and caprine SOLD1 mRNAs have 303 bp open reading frames and encode for deduced SOLD1 proteins with 100 amino acids, including a 22-aa-long signal peptide at the N-terminal. Both of the SOLD1 amino acid sequences have high similarities with the bovine sequence. Both SOLD1 mRNAs were also expressed in TMCs of cotyledons and intercotyledonary membranes. The mature SOLD1 proteins were localized in the mesenchymal villi of cotyledons after secretion. Bovine, ovine and caprine SOLD1 affected gene expression in mesenchymal fibroblasts *in vitro*; nucleoredoxin expression was upregulated and BCL2-like 13 was downregulated. Thus, we suggest that SOLD1 acts as a modulator of cell proliferation and apoptosis.

**Conclusion:**

Expressing cells and protein localization of SOLD1 coincided among the three ruminants. SOLD1 participated in regulating nucleoredoxin and BCL2-like 13 expression in chorionic fibroblasts. SOLD1 is produced specifically in the cotyledons and intercotyledonary membranes in ruminants and appears to be involved in the construction of the ruminant placenta.

## Background

We recently reported bovine secreted protein of Ly-6 (lymphocyte antigen-6, Ly-6/urokinase-type plasminogen activator receptor, uPAR) domain 1, SOLD1, as a lining protein that might participate in the formation of cotyledonary villi [1]. Ruminants such as cattle, sheep and goats, have a cotyledonary placenta. Here we aimed to explore the expression of SOLD1 in sheep and goat placentas and to clarify its roles in the ruminant placenta. We examined gene regulation of chorionic fibroblasts cultured with SOLD1 *in vitro*. We also compared SOLD1 protein characterization and roles among sheep, goats and cattle.

The Ly-6 domain is a 70-100 amino acid (aa) long protein characterized by a conserved pattern of 8-10 cysteine residues with a defined pattern of disulfide bonding [2,3]. The Ly-6 superfamily comprises membrane type glycosylphosphatidylinositol (GPI)-anchored proteins and secreted proteins [2,4]. The secreted members lack a GPI-anchor, such as the secreted LY6/PLAUR domain containing 1 (SLURP1) [4], Ly6/neurotoxin 1 (LYNX1, SLURP2) [5,6], acrosomal vesicle protein 1 (ACRV1, SP10) [7,8], protein expressed in prostate and testis (PATE) [9-11] and secreted seminal vesicle Ly6 protein (Sslp-1) [12]. Genes for these proteins are found mainly in male reproductive tissues, such as the prostate, testis and spermatozoa. In contrast, bovine SOLD1 is exclusively found in female reproductive tissues and was confirmed in the placenta and the intercotyledonary membranes [1]. No expression of SOLD1 orthologs has been reported in human or mouse placentas. There appears to be a Ly-6 domain protein that is essential for placental formation in the ruminant.

## Results

### Characterization of mRNA and deduced protein sequences of ovine and caprine SOLD1

**Figure 1:**
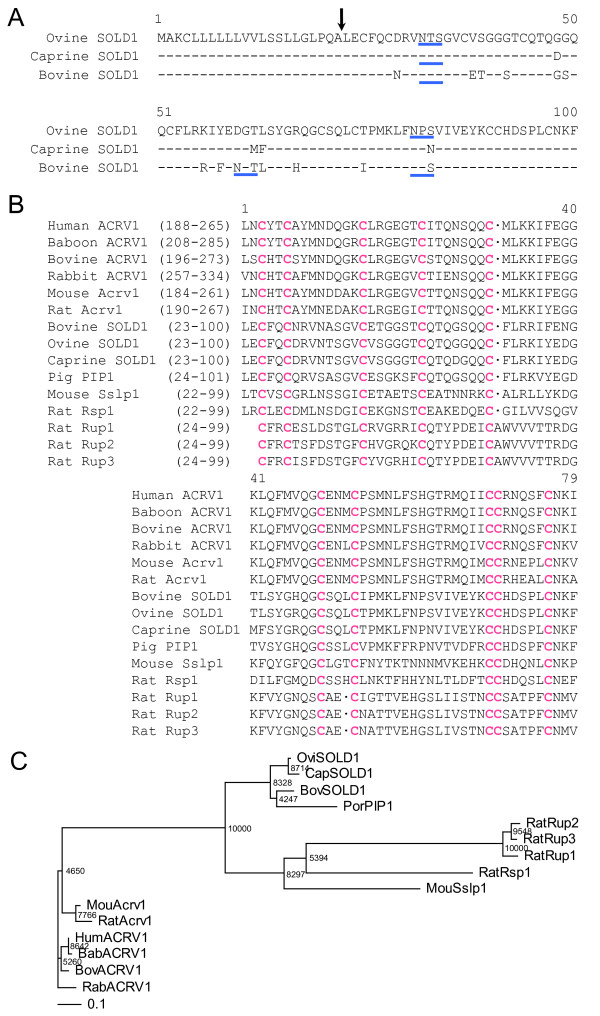
**Properties of SOLD1 amino acid (aa) sequences.** (A) Comparison of amino acid (aa) sequencesbetween ovine (ov) SOLD1, caprine(ca) SOLD1 and bovine (bo) SOLD1. Residues identical among the three SOLD1s are shown by hyphens. The aa sequences were aligned using ClustalW 1.83 on the DNA Data Bank of Japan (DDBJ) web site http://clustalw.ddbj.nig.ac.jp/top-e.html. The arrow indicates the putative primary cleavage site of the signal peptide of each SOLD1. The potential *N*-glycosylation sites are underlined in blue. (B) Comparison of aa sequences between ruminant SOLD1s and the phylogenetically close Ly-6 domain in each proteins selected by ProDom software (see Materials and Methods). Pink characters show residues identical in the Ly-6 domains. The sequence gaps are shown by dots. (C) Phylogenetic tree of mature sheep and goat SOLD1 proteins and the Ly-6 domain regions of some secreted Ly-6 superfamily members. The tree was constructed using TreeView following the alignment of protein sequences given by the ClustalW 1.83 algorithm. The numbers at the base of each branch division represent bootstrap values after 10,000 repeats. The scale bar shows the evolutionary distance between aa sequences estimated using the Kimura method [27]. The scale bar represents 0.1 aa replacements per amino acid site. Key: Bab, baboon; Bov, Bovine; Cap, Caprine; Hum, Human; Mou, Mouse; Ovi, Ovine; Rab, Rabbit.

We cloned cDNA sequences for ovine (*ov*) and caprine (*ca*) *SOLD1 *from placentomes and identified two 303-bp open reading frame cDNA sequences as *ovSOLD1 *and *caSOLD1*, respectively. The sequence deduced from *SOLD1 *cDNAs had 100 aa. The *N*-terminal 22-aa-long regions of both SOLD1 proteins were rich in hydrophobic aa residues characteristic of a signal peptide (Figure 1A). The *SOLD1 *mRNA and deduced mature regions of aa sequences in the three ruminants (ovine, caprine and bovine) were highly homologous at 81-95% (Figure 1A, Table 1). The sequences had 10 cysteines (Cys) and this configuration was identified in all three proteins (Figure 1B). There was a range of 31-71% homology among SOLD1s with rat urinary proteins (Rup-1, Rup-2, Rup-3), rat spleen protein-1 (Rsp-1), porcine protein-1 (PIP-1), mouse Sslp-1 and the *C*-terminal regions of ACRV1 from some species, using domain alignment on data from the DNA Data Bank of Japan (DDBJ) web site http://clustalw.ddbj.nig.ac.jp/top-e.html (Figure 1B and Table 1). Although low homologies in the aa sequences were demonstrated between the Ly-6 domains, the characteristic Cys configuration was intact among all Ly-6 domains in these proteins (Figure 1B). The numbers of potential *N*-glycosylation sites (Asn-X-Ser/Thr) of each ruminant SOLD1 differed (Figure 1A). Thus, ovSOLD1 had two consensus sequences at positions 32-34 and 81-83. By contrast, caSOLD1 had only one consensus sequence at position 32-34 and bovine (bo) SOLD1 had three consensus sequences at positions 32-34, 60-62 and 81-83. Phylogenetic analysis showed that the ovine, caprine and bovine SOLD1 proteins and porcine PIP-1 were phylogenetic neighbors (Figure 1C). We have submitted these sequences to the DDBJ and the DDBJ/GenBank accession Nos. are AB297496 (sheep) and AB297497 (goat).

**Table 1:** Amino acid similarity between SOLD1 and Ly-6 domain of related molecules

Similarity (%)	ovSOLD1	caSOLD1
ovSOLD1	---	95
caSOLD1	95	---
boSOLD1	86	81
poPIP1	71	69
moSslp1	38	41
ratRSP1	32	33
ratRUP1	32	31
ratRUP2	33	33
ratRUP3	33	33
huACRV1	42	41
baACRV1	42	41
boACRV1	41	40
rabACRV1	38	37
moAcrv1	42	41
ratAcrv1	41	40

Key: bo, bovine; po, porcine; mo, mouse; hu, human; ba, baboon; rab, rabbit.

### SOLD1 gene expression

**Figure 2:**
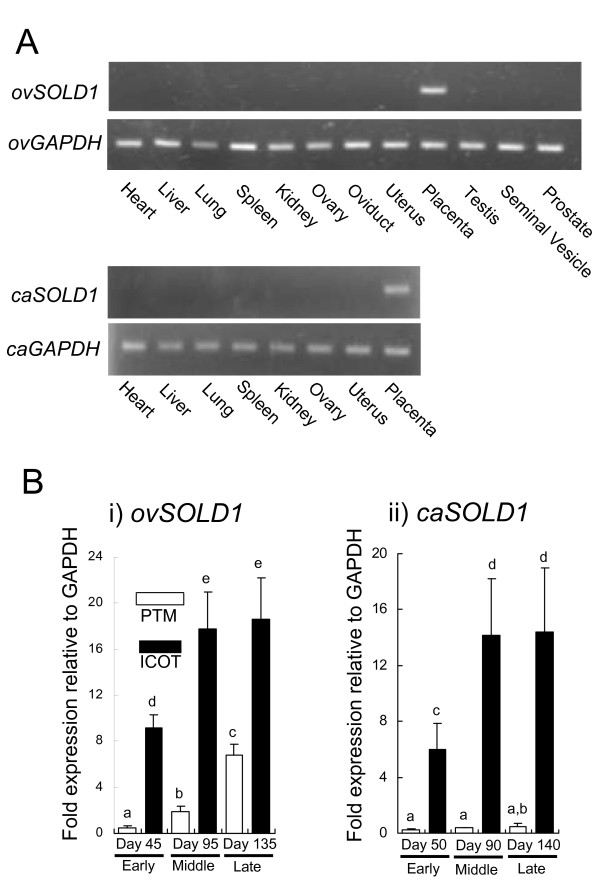
**Expression of ovSOLD1 and caSOLD1 mRNA. **(A) Expression of *ovSOLD1 *and *caSOLD1 *mRNA in ovine and caprine tissues, respectively. Heart, liver, lung, spleen, kidney, ovary, oviduct, uterus, placenta, testis, seminal vesicle and prostate were used for reverse transcription polymerase chain reaction (RT--PCR) amplification in sheep tissues. Heart, liver, lung, spleen, kidney, ovary, uterus and placenta were used for RT--PCR amplification in goat tissues. Ovine and caprine placental tissues taken at days 45 and 50 of gestation were used, respectively. *GAPDH *expression levels in ovine and caprine tissue are presented as loading controls. (B) Expression of *SOLD1 *in ruminant placental tissues by quantitative real-time PCR analysis. i) *ovSOLD1 *expression during the early to late stages of pregnancy. Ovine samples were recorded as Day 45PTM, Day 45ICOT, Day 95PTM, Day 95ICOT, Day 135PTM and Day 135ICOT, respectively. ii) *caSOLD1 *expression during the early to late stage of pregnancy. Caprine samples were recorded as Day 50PTM, Day 50ICOT, Day 90PTM, Day 90ICOT, Day 140PTM and Day 140ICOT, respectively. PTM and ICOT refer to placentome and intercotyledonary membrane, respectively. Expression levels of these mRNAs were normalized to that of *GAPDH *measured in the corresponding RNA preparation. Values are shown as the mean ± SEM. Values with different letters are significantly different (*P *< 0.05).

Figure 2A depicts the tissue distributions of ovine and caprine *SOLD1 *amplified by reverse transcription polymerase chain reaction (RT-PCR). The mRNA appeared only in the placenta in both species. In the sheep, no expression was detected in the heart, liver, lung, spleen, kidney, ovary, oviduct, uterus, testis, seminal vesicle or prostate. In the goat, no expression was detected in the heart, liver, lung, spleen, kidney, ovary, or uterus. Quantification of *ovSOLD1 *expression is depicted in Figure 2B(i). In the placentome (PTM), the *ovSOLD1 *expression level on day 95 of gestation was higher than on day 45 and the expression level on day 135 was higher than on day 95. In the intercotyledonary membrane (ICOT), the expression on day 95 was higher than on day 45 and remained similar on day 135. There was higher *ovSOLD1 *expression in the ICOT than at the PTM in each stage of gestation (Figure 2B(i)). In the caprine PTM, the *caSOLD1 *expression intensity was similar during the early, middle and late stages of gestation. In the caprine ICOT, the expression level on day 90 was higher than on day 45 and remained similar on day 140. Levels of *caSOLD1 *in the ICOT were much higher than in the PTM at each stage of gestation (Figure 2B(ii)). These patterns of expression were common to both species.

**Figure 3:**
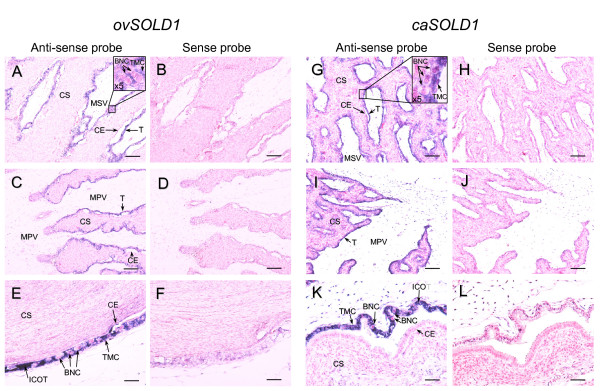
***In situ* hybridization of *SOLD1* mRNA in ovine and caprine placentomes**. (A--F) Messenger RNA localization of *SOLD1 *in ovine placentomes on day 45 of gestation. *ovSOLD1 *mRNA was detected in each frame region by *in situ *hybridization. (A, C and E) Digoxigenin (DIG)-labeled anti-sense cRNA probes were used. (B, D and F) DIG-labeled sense cRNA probes were used. (G--L) Messenger RNA localization of *SOLD1 *in caprine placentomes on day 50 of gestation detected by *in situ *hybridization. (G, I and K) DIG-labeled anti-sense cRNA probes were used. (H, J and L) DIG-labeled sense cRNA probes were used. Key: CE, caruncular epithelium; CS, caruncular stroma; T, trophoblast; TMC, trophoblast mononucleate cell; BNC, trophoblast giant binucleate cell; MPV, mesenchyme of primary villi; MSV, mesenchyme of secondary villi. ICOT, intercotyledonary membrane. Scale bars = 100 μm (A--D and G--J) and 50 μm (E, F, K and L).

We determined *SOLD1 *mRNA localization by *in situ *hybridization in ovine and caprine placentomes at days 45 and 50 of gestation in sheep and goats, respectively (Figure 3). Digoxigenin (DIG)-labeled *ovSOLD1 *and *caSOLD1 *anti-sense RNA specifically detected the mRNA in each species in trophoblast mononucleate cells (TMCs) in cotyledons and in intercotyledonary membranes. There was no expression in giant binucleate cells (BNCs). No *SOLD1 *mRNA was found in the maternal tissues (caruncles and intercaruncular regions in the endometrium). We detected no significant signal with the all sense probes.

**Figure 4:**
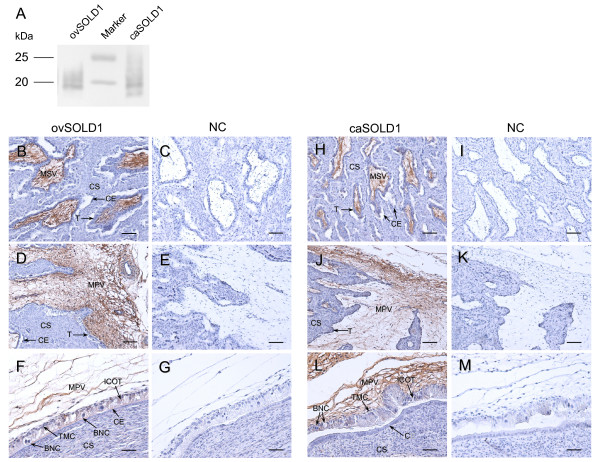
**Western blotting and Immunohistochemistry of SOLD1**. (A) Western blot analysis of recombinant SOLD1 proteins. Purified ovSOLD1 and caSOLD1 (1 ng each) were loaded onto separate lanes. The proteins were separated by SDS--PAGE and specific proteins were detected by western blot analysis using anti-boSOLD1 antibody. (B--G) Protein localization of SOLD1 in ovine placentomes on day 45 of gestation. (B, D and F) The ovSOLD1 protein was detected by immunohistochemistry. A custom-made anti-boSOLD1 antibody was used. (C, E and G) Negative control (NC) using rabbit pre-immune serum instead of the primary antibody. (H--M) Localization of SOLD1 protein in caprine placentomes on day 50 of gestation. (H, J and L) The caSOLD1 protein was detected by immunohistochemistry using a custom-made anti-boSOLD1 antibody. (I, K and M) (NC using rabbit preimmune serum instead of the primary antibody. The key to abbreviations is as in Figure 3. Scale bars = 100 μm (B--E and H--K) and 50 μm (F, G, L and M).

We confirmed that the anti-boSOLD1 antibody was bound to purified recombinant ovSOLD1 and caSOLD1 using western blotting (Figure 4A). The results of immunohistochemistry on ovine and caprine placentomes using the anti-boSOLD1 antibody are shown in Figure 4. Intense staining for SOLD1 was observed in the mesenchymal areas of stem (primary) and branch (secondary) villi. TMCs, --the mRNA-producing cells--were poorly stained. No specific staining was detected in caruncular or intercaruncular endometrium. The staining characteristics were similar in both species (Figure 4).

### Gene regulation of bovine chorionic fibroblasts (BCFs) by SOLD1

**Figure 5:**
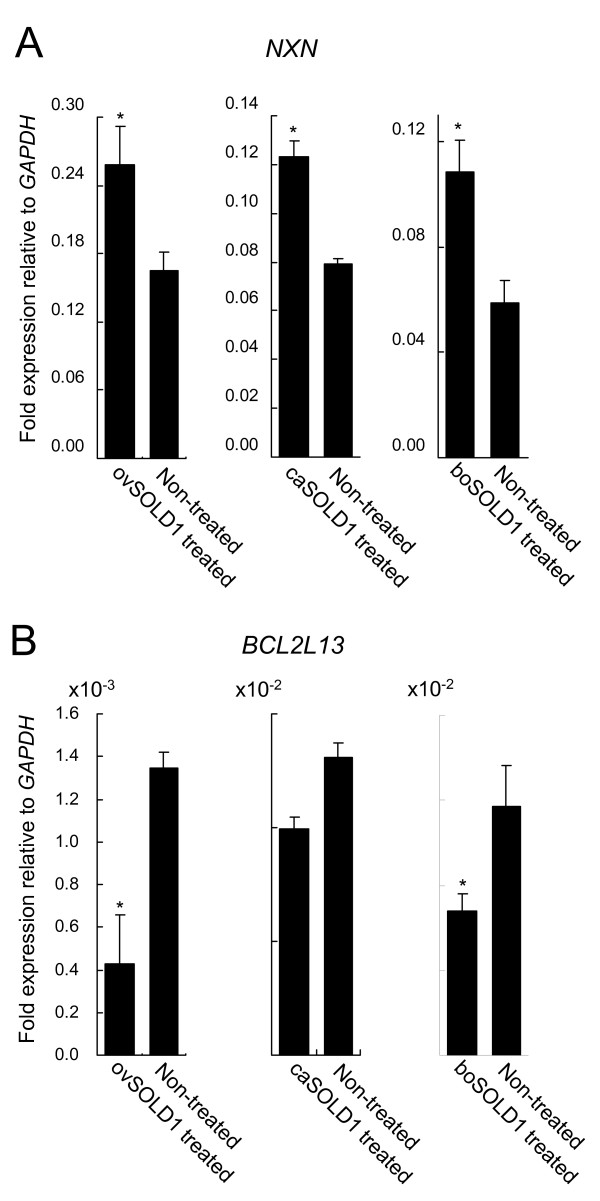
**Differences in gene expression patterns between bovine chorionic fibroblasts (BCFs) treated with and without SOLD1**. Expression levels were measured by real-time quantitative RT--PCR. (A) Nucleoredoxin (*NXN*) expression. (B) BCL2-like 13 (*BCL2L13*) expression. Expression levels of these mRNAs were normalized to that of *GAPDH *measured in the corresponding RNA preparation. Values are shown as the mean ± SEM; *P < 0.05.

We investigated differences in the expression patterns of the genes for nucleoredoxin (*NXN*) and BCL2-like 13 (BCL-Rambo, *BCL2L13*), in BCFs following treatment with ovSOLD1, caSOLD1 and boSOLD1 (Figure 5). *NXN *expression was upregulated by SOLD1 treatment (1.6-fold, *P *< 0.05 by ovSOLD1 treatment, 1.6-fold, *P *< 0.05 by caSOLD1 treatment and 1.8-fold, *P *< 0.05 by boSOLD1 treatment). *BCL2L13 *expression was downregulated by SOLD1 treatment (0.32-fold, *P *< 0.05 by ovSOLD1 treatment and 0.57-fold, *P *< 0.05 by boSOLD1 treatment). ovSOLD1 and boSOLD1 significantly regulated the expression levels of these genes. However, no significant differences were detected in BCL2L13 expression levels in case of the caSOLD1 treatment.

## Discussion

The *SOLD1 *genes are highly homologous among sheep, goats and cattle, showing the general similarity of the Ly-6 domain superfamily (Figure 1). Although the overall cross-species homology was not high for multiply aligned polypeptides, the characteristic Cys configuration was seen consistently. These genes also encode for some potential *N*-glycosylation sites. We therefore predict that these molecules have evolved from a common phylogenetic origin. Currently, it is hard to tell whether these genes and their products have any common functions, because Ly-6 superfamily genes have been detected in various tissues. *ACRV1*, which resembles *SOLD1 *structurally, is a spermatid-specific gene in several species [7,8,13,14]. Mouse *Sslp-1 *is also a spermatid-specific gene [12]; rat *Rup-1*, *Rup-2 *and *Rup-3 *are expressed in urinary organs and rat *Rsp-1 *is expressed in the spleen [15]. *SOLD1 *was mainly expressed in placental tissues in these ruminants (Figure 2). Expression of *PATE-P *and -*Q *have been reported in human placental tissue [11]. However, the aa sequences of these proteins do not show high homology with SOLD1 [1,7,8,11,12,15].

Although both *ovSOLD1 *and *caSOLD1 *mRNA were expressed in TMCs (Figure 3), their encoded proteins were detected in mesenchymal villi (Figure 4). These expression features of the genes and proteins are identical to that found in the bovine [1]. We predict that the SOLD1 protein must have specific functions for ruminants, because no homologous protein has been found in the human or mouse to date. The cotyledonary mesenchyme is mainly composed of extracellular matrix, mesodermal fibroblasts and vascular endothelial cells. We have reported that boSOLD1 is secreted to the basolateral surface in trophoblast and is mainly bound to a tetrapeptide of type I collagen [1]. It has been suggested that SOLD1 is an essential protein for cotyledonary formation. However, the physiological role of SOLD1 is still unknown. Here, we investigated the effect of SOLD1 on gene expression in BCFs. NXN is a thioredoxin (TRX) family protein and mainly suppresses the Wnt-beta-catenin pathway [16]. Its effect is to suppress cell proliferation. BCL2L13 is a pro-apoptotic BCL2 family member [17] and its expression promotes apoptosis. We found here that *NXN *expression was upregulated in BCFs by exposure to ovSOLD1, caSOLD1 or boSOLD1 and this is likely to suppress their proliferation. In contrast, *BCL2L13 *was downregulated in BCFs by exposure to ovSOLD1 or boSOLD1 and this is likely to suppress apoptosis. We suggest that SOLD1 acts as a modulator of proliferation and apoptosis in chorionic fibroblasts. We also expect that SOLD1 will show a similar role among the ruminants. It is known that Lypd6, SLURP1, LYNX1 and the PATE family are ligands of the nicotinic acetylcholine receptor (nAChR) in the secreted Ly-6 family of several tissues [6,11,18,19]. This receptor regulates sodium and calcium ion channels. If SOLD1 acts as a ligand of nAChR it is likely that the expression levels of *NXN *and *BCL2L13 *are regulated via such ion channel pathways.

The trophoblast cells of the ruminant placenta are divided into BNCs and TMCs [20]. BNCs account for approximately 20% of the total trophoblast cell population [20]. They are directly involved in modification of the uterine epithelium beginning at implantation and continuing until term. BNC products, such as placental lactogen, pregnancy-associated glycoproteins and steroid hormones, are not restricted in terms of their direction of secretion [21-23]. Hence, the BNCs play a pivotal role in fetal-maternal communication in ruminants. TMCs are a source of interferon-tau (IFNT), which participates in the recognition of gestation during the peri-implantation period in ruminants. Thus the production of IFNT is a specific role of TMCs. However, other roles of placental TMCs are obscure. One function might be the production of SOLD1 proteins to develop villi. The matrix metalloproteinases, MMP2 and MMP9, are also produced by ruminant TMCs [24]. In the human syncytiotrophoblast, MMP2 and MMP9 are secreted to the basolateral surface of the cells and modify the basement membrane [25]. This is similar to the secretion property of SOLD1 [1]. TMCs have polarity and this might be related to the production of a protein involved in determining the direction of secretion.

## Conclusion

In conclusion, we have identified two sheep and goat secreted proteins, ovSOLD1 and caSOLD1, each composed only of the Ly-6 domain and a signal peptide. Both types of *SOLD1 *mRNA appeared in TMCs throughout gestation and their proteins were localized in the mesenchyme of primary and secondary placental villi. Phylogenetic configuration, expression and protein localization were similar among the ruminant SOLD1 proteins and bovine SOLD1 orthologs are found in the sheep and goat. As for the physiological role of SOLD1, which is unknown for cattle, we examined the effects of SOLD1 on BCFs. SOLD1 might be one of the main factors for modulating and establishing the villous mesenchyme in ruminants through the regulation of *NXN *and *BCL2L13 *expression in chorionic fibroblasts.

## Methods

### Animals and tissue collection

Placental tissues for cDNA cloning, mRNA quantitative expression, *in situ *hybridization and immunohistochemistry were collected from Corriedale sheep and Japanese Saanen goats. The animals were maintained in the ranch of our laboratory. For sheep, PTM and ICOT tissues were collected on days 45 (*n *= 3 animals), 95 (*n *= 3) and 135 (*n *= 3) of gestation after natural mating (designated as day 1 of pregnancy). For the goats, placental tissues were collected on days 50 to 52 (*n *= 4 animals), 86 to 92 (*n *= 4) and 135 to 139 (*n *= 3) after artificial insemination (designated as day 1 of pregnancy). These tissues were also divided into PTM and ICOT portions. Heart, liver, lung, spleen, kidney, ovary, oviduct and uterus tissues were collected from a non-pregnant ewe as reference material. Testis, seminal vesicle and prostate tissues were collected from a ram. Heart, liver, lung, spleen, kidney, ovary and uterus tissues were collected from a non-pregnant goat.

Samples were stored at -80°C before RNA extraction. Some placentomes were fixed in 3.7% formaldehyde in phosphate buffered saline (PBS) at pH 7.4, embedded in paraffin wax and stored at 4°C for *in situ *hybridization and immunohistochemistry. All procedures were carried out in accordance with the ethical guidelines approved by the Animal Ethics Committee of the National Institute of Agrobiological Sciences for the use of animals.

### Cloning of the coding sequence region of SOLD1 cDNA

The coding sequence regions of *ovSOLD1 *and *caSOLD *cDNAs were isolated from cotyledonary tissues using RT-PCR amplification. In brief, total RNA was isolated from sheep or goat placentomes using ISOGEN (Nippon Gene, Toyama, Japan). DNase treatment for the genomic DNA removal was performed using TURBO DNA-free kits (Ambion, Austin, TX, USA). The total RNA in a reaction mixture was used for RT and template cDNA synthesis using oligo(dT) primers and Superscript III reverse transcriptase (Invitrogen, Carlsbad, CA, USA) at 50°C for 50 min. We performed PCR with common *SOLD1*-specific primers used for ruminants (Table 2). The *SOLD1 *primers were designed based on the hypothetical bovine protein LOC100125878 sequences (GenBank reference accession number NM_001105478). The products were sequenced using an ABI Prism 370 automatic sequencer (Applied Biosystems, Foster City, CA, USA) after cloning into a pGEM-T Easy vector (Promega, Madison, WI, USA).

**Table 2:** Oligonucleotide primers used for cDNA cloning or RT--PCR analysis

Gene (accession number)	Primer	Sequence	Position*
*ovSOLD1 *and *caSOLD1*	Forward	5'--TCCAGAGATGGCTAAGTGCCTT--3'	50--71
(NM_001105478)	Reverse	5'--GAGTTGGACATGACTGAGCCAC--3'	453--432
*ovGAPDH*	Forward	5'--AAGGCCATCACCATCTTCCA--3'	78--97
(AF030943)	Reverse	5'--AGGTCAGATCCACAACGGACA--3'	603--583
*caGAPDH*	Forward	5'--GACCCCTTCATTGACCTTCAC--3'	1--21
(AJ431207)	Reverse	5'--TCATAAGTCCCTCCACGATGC--3'	424--404

* The nucleotide position in each accession number

### Multiple alignment of the deduced protein sequences

The deduced ovSOLD1, caSOLD1 and boSOLD1 protein sequences were aligned using the multiple alignment software ClustalW 1.83 on the DDBJ web site http://clustalw.ddbj.nig.ac.jp/top-j.html using the neighbor-joining (NJ) method with Kimura distance [26,27]. Domain retrieval of the SOLD1 protein was performed using the ProDom web site http://prodomweb.univ-lyon1.fr/prodom/current/html/home.php. Ruminant SOLD1 and some secreted Ly-6 domain sequences were also aligned using ClustalW 1.83. TreeView software was used to display the phylogenetic tree [28]. The values represent bootstrap scores for 10,000 trials, indicating the credibility of each branch.

### RT-PCR

The tissue distributions of *ovSOLD1 *and *caSOLD1 *mRNA expression were studied using RT-PCR with *GAPDH *as a positive control [29,30]. The total RNA in a reaction mixture was used for reverse transcription and template cDNA synthesis using oligo(dT) primers (Table 2) and Superscript III reverse transcriptase (Invitrogen) at 50°C for 50 min. Each PCR contained the cDNA template, primers, autoclaved MilliQ water and AmpliTaq Gold PCR master mix (Applied Biosystems). Amplification conditions were an initial denaturation step at 95°C for 10 min, 26 cycles of denaturation at 95°C for 30 sec, annealing at 57°C for 30 sec, extension at 72°C for 1 min and a final extension step at 72°C for 10 min. The PCR products were analyzed by agarose gel electrophoresis and visualized by ethidium bromide staining. The same primers encoding the *SOLD1 *sequence were used for cDNA cloning (Table 2; Tsukuba Oligo Service, Tsukuba, Japan).

### Quantitative real-time RT-PCR (qRT-PCR)

Expression levels of *ovSOLD1 *and *caSOLD1 *were confirmed quantitatively at each stage of gestation by qRT-PCR using the Power SYBR Green PCR master mix (Applied Biosystems). Fifty nanogram aliquots of total RNA were reverse-transcribed into cDNA for 30 min at 48°C using MultiScribe reverse transcriptase with a random primer, dNTP mixture, MgCl_2 _and RNase inhibitor followed by heat inactivation of the reverse transcriptase for 5 min at 95°C. In the SYBR Green assay, primer pairs were designed using the Primer Express software program (Applied Biosystems). The primers for each gene are listed in Table 3. Cycling conditions included initial incubation at 50°C for 2 min and 95°C for 10 min, followed by 40 cycles of 95°C for 15 s and 60°C for 1 min. The resulting relative increase in reporter fluorescent dye emission was monitored in real time using an Mx3000P QPCR system (Stratagene, La Jolla, CA, USA). The relative differences in the initial amounts of each cDNA species were determined by comparing the threshold cycle (C_T_) values. Standard curves for each gene were generated by serial dilution of each plasmid containing the corresponding cDNA to quantify the mRNA concentrations. We confirmed the dissociation curve for detecting the SYBR Green-based objective amplicon, because SYBR Green also detects any double-stranded DNA including primer dimers, contaminating DNA and PCR products from misannealed primers. Thus, contaminating DNA or primer dimers would show up as a peak separate from the desired amplicon peak. The expression ratio of each gene to *GAPDH *mRNA was calculated to adjust for variations in the qRT-PCR reaction. All values are presented as the mean ± SEM. Replications of qRT-PCR data were performed on samples from three or four animals and technical duplicates were done from each animal sample (six or eight data points in total). Statistical analysis was performed using one-way ANOVA followed by the Tukey-Kramer multiple-comparison test. Differences were considered significant at *P *< 0.05.

**Table 3:** Oligonucleotide primers used for qualitative real-time PCR analysis

Gene (accession number)	Primer	Sequence	Position*
*ovSOLD1*	Forward	5'--GGAGGCACCTGCCAGACTCA--3'	128--147
(AB297496)	Reverse	5'--AAAGCGTGCCATCTTCGTAG--3'	197--178
*caSOLD1*	Forward	5'--GGAGGCACCTGCCAGACTCA--3'	128--147
(AB297497)	Reverse	5'--AAAACATGCCATCTTCGTAG--3'	197--178
*ovGAPDH*	Forward	5'--GCCATCACCATCTTCCAGGA--3'	81--100
(AF030943)	Reverse	5'--CCACGTACTCAGCACCAGCA--3'	150--131
*caGAPDH*	Forward	5'--GCCATCACCATCTTCCAGGA--3'	115--134
(AJ431207)	Reverse	5'--CCACGTACTCAGCACCAGCA--3'	184--165
*boNXN*	Forward	5'--TCCTAGTGGAGTCCTACCGGAA--3'	715--736
(NM_001102136)	Reverse	5'--CCTGTCCGCACTAACAAAGATG--3'	788--767
*boBCL2L13*	Forward	5'--TCGGGAATGTACACTGGAGACC--3'	592--613
(NM_001078082)	Reverse	5'--GTAGCAAAATCAGAGGCACCAA--3'	665--644
*boGAPDH*	Forward	5'--AAGGCCATCACCATCTTCCA--3'	178--197
(U85042)	Reverse	5'--CCACTACATACTCAGCACCAGCAT--3'	253--230

* The nucleotide position in each accession number

### *In situ *hybridization

*ovSOLD1 *and *caSOLD1 *cDNA were used as templates for hybridization probe synthesis. DIG-labeled antisense- and sense-complementary RNA probes were prepared using DIG RNA labeling kits (Roche Diagnostics, Basel, Switzerland) and the cloned coding sequence region of *ovSOLD1 *or *caSOLD1 *cDNA in plasmids as described [29,30]. Placentomes were sectioned at 7 μm and *in situ *hybridization was performed using an automated Ventana HX System Discovery with a RiboMapKit and BlueMapKit (Ventana, Tucson, AZ, USA). Briefly, ovine and caprine sections were hybridized with DIG-labeled probes in RiboHybe (Ventana) hybridization solution at 68°C (for *ovSOLD1*) or 61°C (for *caSOLD1*), for 6 h. The sections were washed three times in RiboWash (Ventana) (68°C for *ovSOLD1 *or 61°C for *caSOLD1*, 6 min each) after hybridization and fixed in RiboFix (Ventana) (37°C, 10 min). The hybridization signals were then detected using anti-digoxin monoclonal antibody-biotin conjugates (Sigma-Aldrich, St Louis, MO, USA). Counterstaining was done with nuclear fast red (Ventana). The hybridized slides were observed using a Leica DMRE HC microscope (Leica Microsystems, Wetzlar, Germany) equipped with a DS-Fi1 digital camera and a DS-L2 control unit (Nikon, Tokyo, Japan).

### Production and purification of recombinant proteins

The ovSOLD1, caSOLD1 and boSOLD1 sequences encoding the mature protein region (aa 23-100), which included the FLAG and 6× His epitope tag sequences, were inserted into a pFLAG-CMV-3 vector (Sigma-Aldrich). The constructed plasmid was transfected into HEK 293 cells using FuGENE 6 (Roche). Stably transfected HEK 293 cells were adapted to suspension culture in a spinner flask using 293 SFM II medium (Gibco, Invitrogen) and cultured in an atmosphere of 5% CO_2 _in air at 37°C for three days. The medium was separated from cells by centrifugation.

Recombinant FLAG-tag and 6× His-tag fusion proteins were purified using the 6× His-tag portion. Approximately 1 l of conditioned medium was processed at a time. Medium to which 1 ml Ni Sepharose 6 Fast Flow (Amersham Biosciences, Buckinghamshire, UK) was added was mixed and equilibrated with 20 mM sodium phosphate buffer, pH 8.0, containing 300 mM NaCl and 20 mM imidazole. Only the 6× His-tag proteins bind to the Ni Sepharose 6 Fast Flow carrier. The medium with carrier was chromatographed on a PD-10 column (Amersham Biosciences). The fractions with carrier were washed with 20 mM imidazole. The fractions were eluted with 250 mM imidazole.

### Immunohistochemistry

Immunohistochemistry was performed using an automated Ventana HX System Discovery with DabMapKit reagents (Ventana). Custom-made anti-bovine SOLD1 (anti-boSOLD1) antibody was used for immunohistochemistry. The mature boSOLD1 region (aa 23-100) involving the epitope was used for raising antigen in rabbits (Operon Biotechnology, Tokyo, Japan) [1]. The antibody's quality and specificity was confirmed with the purified recombinant ovine and caprine SOLD1 protein, respectively, using western blotting analysis. For immunohistochemistry, the sections were incubated with anti-boSOLD1 antibody and preimmune serum (negative control) at a dilution of 1:100 in Ab Diluent (Ventana) for 4 h. The sections were then washed and incubated with an anti-rabbit IgG-Biotin conjugate at a dilution of 1:500 (Sigma-Aldrich) for 1 h. Immunoreactive signals were detected using streptavidin-horseradish peroxidase (HRP) and diaminobenzidine (DabMapKit, Ventana). Counterstaining was done with hematoxylin and bluing reagent (saturated lithium carbonate solution). After treatment, the sections were observed using a Nikon Eclipse E800 photomicroscope equipped with a DS-Fi1 digital camera and a DS-L2 control unit (Nikon, Tokyo, Japan).

### SOLD1 treatment of bovine chorionic fibroblasts (BCFs) and gene expression analysis

BCFs were prepared as follows. Bovine cotyledons were collected from a pregnant cow (Japanese Black) at day 64 after insemination. The cotyledonary tissues were dissected aseptically from the surrounding tissues. Small pieces of tissues were incubated for 1 h at 37°C in an atmosphere of 5% CO_2 _in air in Dulbecco's Modified Eagle Medium (DMEM)/Ham's Nutrient Mixture F12 (F12) 1:1 medium mixture supplemented with 20% fetal bovine serum (FBS) (DFF20 medium) containing 0.1% collagenase (Wako, Osaka, Japan). The cell suspension was centrifuged at 1000 *g *for 10 min and washed twice with DFF20 medium. The cells were incubated in T-25 flasks (BD Biosciences, Franklin Lakes, NJ, USA) for 1 h at 37°C in DFF20 The cell suspension was moved to type-I collagen coated T-25 flasks (BD Biosciences) and incubated for 10 days at 37°C in DFF20. The medium was replaced twice during this period. The medium was removed and the cells were washed with PBS. The cells were then treated with trypsin-EDTA (0.05% trypsin and 0.53 mM EDTA; Gibco, Invitrogen). The suspended fibroblasts were cultured and the remaining adhesive epithelial cells were discarded. The fibroblasts were established based on trypsin sensitivity over twelve passages.

Cell synchronization of the BCFs was performed prior to ovSOLD1, caSOLD1 or boSOLD1 treatment. Thymidine (2 mM final concentration) was added to the cell suspension (density 2 × 10^5^) in DMEM/F12 1:1 supplemented with 10% FBS (DFF10 medium) and the cells were incubated for 12 h at 37°C under 5% CO_2 _in air. The cells were placed in thymidine-free DFF10 with the same atmosphere to exit from the S phase after being washed with FBS free DMEM/F12 medium. Mimosine (400 μM final concentration) was added to the cell suspension in DFF10 medium and the cells were incubated for 12 h further at 37°C under 5% CO_2 _in air. The cells were arrested at the G1/S cell cycle boundary prior to each SOLD1 treatment [31]. Eight T-25 flasks containing the synchronized cells were prepared for each SOLD1 treatment and negative controls. The ovSOLD1, caSOLD1 or boSOLD1 (final concentration 10 ng/ml in DMEM/F12) was added to six of the flasks and incubated for 2 d at 37°C under 5% CO_2 _in air. Negative controls were incubated without SOLD1 in the same conditions. RNA was isolated from all cells using ISOGEN (Nippon Gene). Expression levels of *NXN *and *BCL2L13 *were confirmed quantitatively by qRT-PCR as above. Primer sequences for bovine *NXN*, *BCL2L13*, *and GAPDH *are listed in Table 3. Statistical analysis was performed using Student's *t*-tests and differences were considered significant at *P *< 0.05.

## Authors' contributions

KU participated in the design of the study and carried out most of the experiments. TT participated and coordination in the design of the study and performed the recombinant protein and antibody productions. KU, TT, MH, KK and KH collected the tissue samples. MH carried out all animal care. KH participated and coordination in the design of the study and helped to draft the manuscript. All authors read and approved the final manuscript.
